# COULD THE INTESTINAL EPITHELIAL ALTERATIONS PROMOTED BY ROUX-EN-Y GASTRIC BYPASS EXPLAIN HIGHER TENDENCY FOR COLONIC DISEASES IN BARIATRIC PATIENTS?

**DOI:** 10.1590/0102-672020200004e1570

**Published:** 2021-03-23

**Authors:** Eduardo WENDLER, Osvaldo MALAFAIA, Bruno Luiz ARIEDE, Jurandir Marcondes RIBAS-FILHO, Nicolau Gregori CZECZKO, Paulo Afonso Nunes NASSIF

**Affiliations:** 1 Post-Graduation Program in Principles of Surgery, Mackenzie Evangelical College of Paraná, Curitiba, PR, Brazil

**Keywords:** Jejunoileal bypass, Tumor suppressor protein p53, Ki-67 antigen, Obesity, Bypass jejunoileal, Proteína supressora de tumor p53, Antígeno Ki-67, Obesidade

## Abstract

***Background*:**

Intestinal diversions have revolutionized the treatment of morbid obesity due to its viability and sustained response. However, experimental studies suggest, after these derivations, a higher risk of colon cancer.

***Aim*:**

To analyze the histological and immunohistological changes that the jejunojejunal shunt can produce in the jejunum, ileum and ascending colon.

***Method*:**

Twenty-four male Wistar rats were randomly divided into two groups, control (n=12) and experiment (n=12) and subdivided into groups of four. Nine weeks after the jejunojejunal shunt, segmental resection of the excluded jejunum, terminal ileum and ascending colon was performed. Histological analysis focused on the thickness of the mucosa, height of the villi, depth of the crypts and immunohistochemistry in the expression of Ki-67 and p53.

***Results*:**

Significant differences were found between the experiment and control groups in relation to the thickness of the mucosa in the jejunum (p=0.011), in the ileum (p<0.001) and in the colon (p=0.027). There was also a significant difference in relation to the height of the villus in the ileum (p<0.001) and the depth of the crypts in the jejunum (p0.001). The results indicated that there is a significant difference between the groups regarding the expression of Ki-67 in the colon (p<0.001). No significant differences were found between the groups regarding the expression of Ki-67 in the jejunum and ileum. In the P53 evaluation, negative nuclear staining was found in all cases.

***Conclusion*:**

The jejunojejunal deviation performed in the Roux-in-Y gastrojejunal bypass, predispose epithelial proliferative effects, causing an increase in the thickness of the mucosa, height of the villi and depth of the crypts of the jejunum, ileum and ascending colon.

## Graphical abstract

[Fig f1a]

**Figure f1a:**
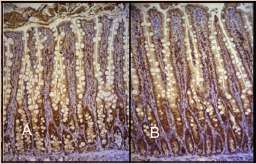
Ki-67 in ascending colon: A) experiment and B) control

[Fig f1as]

**Figure f1as:**
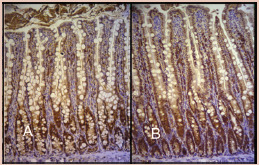
Ki-67 no cólon ascendente: A) experimento e B) controle

## RESUMO

***Racional*:**

As derivações intestinais revolucionaram o tratamento da obesidade mórbida pela sua viabilidade e resposta sustentada. Porém, estudos experimentais sugerem, após estas derivações, risco maior de câncer de cólon.

***Objetivo*:**

Analisar as alterações histológicas e imunoistológicas que a derivação jejunojejunal possa produzir no jejuno, íleo e cólon ascendente.

***Método*:**

Foram utilizados 24 ratos Wistar machos randomicamente divididos em dois grupos, controle (n=12) e experimento (n=12) e subdivididos em grupos de quatro. Nove semanas após a derivação jejunojejunal procedeu-se a ressecção segmentar do jejuno excluso, íleo terminal e cólon ascendente. Análise histológica focou na espessura da mucosa, altura dos vilos, profundidade das criptas e a imunoistoquímica na expressão do Ki-67 e p53.

***Resultados*:**

Foram encontradas diferenças significativas entre os grupos experimento e controle em relação à espessura da mucosa no jejuno (p=0,011), no íleo (p<0,001) e no cólon (p=0,027). Também houve diferença significativa em relação à altura dos vilos no íleo (p<0,001) e profundidade das criptas no jejuno (p<0,001). Os resultados indicaram que existe diferença significativa entre os grupos em relação à expressão do Ki-67 no cólon (p<0,001). Não foram encontradas diferenças significativas entre os grupos em relação à expressão do Ki-67 no jejuno e no íleo. Na avaliação do P53, foi encontrada coloração nuclear negativa em todos os casos.

***Conclusão*:**

O desvio realizado na derivação gastrojejunal em Y-de-Roux, predispõem efeitos proliferativos epiteliais, causando aumento da espessura da mucosa, altura dos vilos e profundidade das criptas do jejuno, íleo e cólon ascendente.

## INTRODUCTION

The intestinal derivations revolutionized the morbid obesity treatment for their feasibility and sustained response. However, experimental studies suggest a higher risk of colon cancer after these derivations. The surgical reduction in the intestinal absorption area produces compensatory hyperplasia in the remaining intestine, which is proportional to the extension of the removed segment. The events underlying this response have been mostly studied in rat intestines, in which the partial enterectomy increases colorectal carcinogenesis. However, intestinal adaptation phenomena occur in humans^6^ ^,^ ^14^ ^,^ ^15^. Disabsorptive procedures allow a bigger quantity of biliopancreatic secretion - normally absorbed by the small intestine - to reach the large intestine^17^ ^,^ ^30^, promoting anomalous situations. The effects of the bile acids on the large intestine carcinogenesis include several factors^16^ ^,^ ^21^ ^,^ ^26^. However, bile acids have an important action in inducing the cell proliferation, which has a critic role in colon carcinogenesis. Bile salts and fat enriched diets influence positively the expression levels of proliferating cell nuclear antigen - PCNA, reflecting a stimulating effect directly in the colonocytes^5^. The levels of colonic crypt cell proliferation are increased in patients who were submitted to jejunoileal shunt for obesity^2^ ^,^ ^3^ ^,^ ^4^. The demonstration that the increase in the fecal bile salts concentration can enhance the colonocytes proliferation is still controversial, as well as the magnitude of the metabolic alteration of these cells after the malabsorptive surgery. Sprague-Dawley rats submitted to this derivation showed 33% increase in crypts depth in the middle third of colons and 25% in the distal colon. After derivation, the middle third crypt cells of the large intestine doubled, whereas they tripled in the distal colon. Existing data demonstrated a sustainable and substantial increase in the cell proliferation levels and adaptation of the large intestine after the jejunoileal shunt^1^ ^,^ ^24^. 

Studies in an animal model suggest an increase in risk of colon cancer. It probably occurs due to the increase of the apoptosis inhibition, crypt cell hyperproliferation and crypt hyperplasia^8^ ^,^ ^12^. As the colonic hyperplasia derives from this set of procedures, a high number of young and middle-aged patients can be at risk of developing large intestine cancer. These findings can, in the future, influence directly the modern surgical practice. 

After experimental jejunojejunal derivation, the present study has the objective of presenting histological and immunochemical alterations that occur in the jejunum, ileum and ascending colon. 

## METHOD

The study was carried out in the Post-Graduation Program in Principles of Surgery from Mackenzie Evangelical College of Paraná, Curitiba, PR, Brazil and it was approved by the institution´s Research Ethics Committee in compliance with the ethical principles of animal experimentation established in the Federal Law 11794 of 10/08/2008. 

Twenty-four male Wistar rats (*Rattus novergicus albinus*, rodentia mamalia) were used, with average weight of 347 g, divided into two groups of 12 animals: control and experimental. Both groups were subdivided into three subgroups of four. The light/dark cycle was maintained artificially in periods of 12 h, the temperature between 18-23º C and the relative humidity was the same as the environment. The animals received water and animal feed (Nuvilab CR1^®^ Nuvital, Colombo, PR) ad libitum.

**FIGURE 1:**
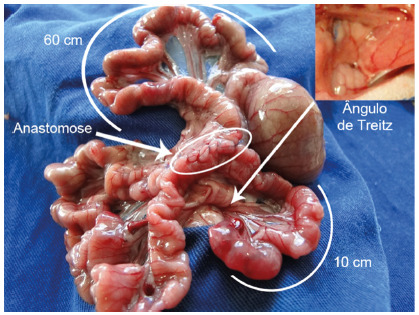
Jejunojejunal derivation by laterolateral anastomosis 10 cm from the duodenojejunal flexure (Treitz) until the laterolateral anastomosis 60 cm from the ileocecal valve

Subcutaneous anesthesia was performed in all the animals using ketamine hydrochloride (50-75 mg/kg) and xylazine (5-10 mg/kg). After abdominal epilation, a median abdominal incision of approximately 3 cm was performed. The small intestine was exposed and measured in its totality. A jejunojejunal derivation was performed by laterolateral anastomosis 10 cm from the duodenojejunal angle and the jejunum segment, localized 60 cm from the ileocecal valve (Figure 1). The single layered intestinal sutures were done using 5-0 polypropylene yarn. After that, the intestinal loops were re-introduced and the cavity was closed by layers. The rats of the control group were not submitted to anastomosis, only to laparotomy and manipulation of the cavity. 

In the post-surgery period, the analgesia was provided by intramuscular application of 0,01 to 0,05 mg/kg of buprenorphine. The death was carried out by carbon dioxide¹² inhalation and after that, the animal was placed in supine position with the paws fixed by elastics. Median re-laparotomy was proceeded by exposition of the intestinal loops. The excluded jejunum, the terminal ileum and the ascending colon were prepared by irrigation of the intestinal lumen with saline solution for removal of the fecal waste. Subsequently, it was instilled 10% formalin fixative solution by flexible catheter. After this preparation, a segmental resection of the excluded jejunum, terminal ileum and ascending colon was performed. In the control group it was performed laparotomy with identification of the intestinal segments and preparation identical to the experimental group for later segmental resection of the proximal jejunum, terminal ileum and ascending colon. 

In the histological study, the sections for morphometric analysis were stained with H&E, analyzing the mucosa thickness, the villi height and the crypt depth. The measurement was performed using the software Dino-Capture 2.0 previously calibrated and three representative areas were measured from each case. Sections for immunohistochemistry were subjected to the following antibodies: p53 - clone SP-5 (Cell Marque, Rocklin, EUA) pre-diluted and incubated for 1 h and Ki-67 - clone SP-6 (BioSB, Santa Barbara, EUA), dilution of 1:500 and incubation of 1 h. As detection system was used MACH4 (Biocare Medical, Concord, CA, USA) with chromogen DAB and counterstain with hematoxylin. Positive and negative external controls were performed together. For the p53 reaction analysis, nuclear stain was considered positive. The histological slides were scanned on panoramic lens (40x) and in negativity case, in 10 fields at 400x. For the Ki-97 reactions it was counted 200 mucosa epithelial cells throughout its thickness and the percentage of positive cells was registered. 

### Statistical analysis

The results obtained in the study were described by means, medians, minimum and maximum values and standard deviations of the three measurements done on each observation unit. For the comparison between the control and experimental group, it was considered the non-parametric Mann-Whitney test. Values of p>0,05 indicated statistical significance. The data was analyzed using the program Statistica v.8.0. 40. In the evaluation of p53, negative nuclear stain was found in all the cases. Therefore, the statistical analysis for this variable was not carried out. 

## RESULTS

### Thickness of the mucosa, villi height and crypt depth

 For each one of these histological variables it was tested the null hypothesis that the mean in the experimental group was equal to the one in the control group vs. the alternative hypothesis of different means in each one.

Significant differences were verified on the mucosa thickness; it was observed that in the three places the mean of the experimental group was higher than the one of the control group in regard to the jejunum (p=0,011), ileum (p=0,001) e colon (p=0,027, Table 1). 

**TABLE 1:** Evaluation of the mucosa thickness of the jejunum, ileum and colon in the experimental and control groups.

Variable mucosa thickness	Group	n	Mean	Median	Mínimum	Maximum	Standard deviation	p
Jejunum	Control	12	477.9	470.3	424.0	588.7	43.0	0.011
	Experimental	12	590.4	506.3	436.0	777.0	126.2	
Ileum	Control	12	417.5	398.8	378.0	515.7	42.3	<0.001
	Experimental	12	528.1	522.7	420.0	53.9.0	52.0	
Colon	Control	12	212.5	205.7	182.7	274.7	26.7	0.027
	Experimental	12	240.9	241.0	158.7	279.7	31.8	

Regarding the villi height, a significant difference was verified when the control group´s ileum (p<0,001) was compared with the higher mean of the experimental group (Table 2) 

**TABLE 2:** Evaluation of the jejunum and ileum villi height in the experimental and control groups.

Variable villi height	Group	n	Mean	Median	Mínimum	Maximum	Standard deviation	p
Jejunum	Control	12	316.8	303.7	275.7	372.3	35.2	0.145
	Experimental	12	351.3	356.8	256.0	444.7	69.9	
Ileum	Control	12	221.1	219.0	185.0	278.0	32.8	<0.001
	Experimental	12	314.8	309.7	240.3	372.3	37.8	

Regarding the crypt depth, a significant difference was verified when the control group´s jejunum (p<0,001) was compared with the higher mean of the experimental group (Table 3) 

**TABLE 3:** Evaluation of the jejunum and ileum crypt depth in the experimental and control groups.

Variable crypt depth	Group	n	Mean	Median	Mínimum	Maximum	Standard deviation	p
Jejunum	Control	12	134.4	125.0	96.3	215.3	30.6	<0.001
	Experimental	12	239.1	232.7	152.3	347.3	59.8	
Ileum	Control	12	196.6	198.0	117.7	251.7	39.1	0.280
	Experimental	12	213.2	219.8	150.0	266.7	34.0	

**FIGURE 2:**
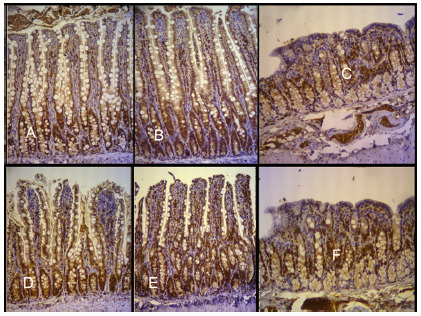
Comparative marking with immunostaining with Ki-67 antibody, with letters representing respectively the experimental and control groups: A and D) excluded jejunum; B and E) terminal ileum; C and F) ascending colon

Considering the expression of Ki-67 in each evaluation place and the differences among the places, it was tested the null hypothesis that the medians were equal in both groups vs the alternative hypothesis for different medians (Table 4, Figure 2). The results indicated that there is a significant difference among the groups regarding the expression of Ki-67 in the colon (p<0,001) with higher average expression from the control group rather than the experimental group (means 49,67 e 40,92 respectively). Significant differences were not found among the groups regarding the expression of Ki-67 in the jejunum and ileum. 

**TABLE 4:** Ki-67 expression in the jejunum, ileum and colon in control and experimental groups

Variable	Group	n	Mean	Median	Minimum	Maximum	Standard deviation	p
Jejunum	Control	12	40.92	39.50	35.00	52.00	4.93	<0.001
	Experimental	12	49.67	49.50	47.00	52.00	1.72	
Ileum	Control	12	83.50	84.50	75.00	89.00	4.64	0.307
	Experimental	12	58.00	85.00	82.00	87.00	1.54	
Colon	Control	12	87.00	86.00	78.00	94.00	4.31	0.142
	Experimental	12	84.92	85.00	83.00	89.00	1.73	

In the p53 evaluation, it was found negative nuclear staining in all cases. Thus, statistical analysis for this variable was not done.

## DISCUSSION

The jejunoileal derivation is a modality of malabsorptive surgery of low complexity when performed in rats. Scudamore, C.H. e Freeman, H.J.^28^ performed jejunoileal derivation anastomosis in 35 Wistar rats 35 cm from the ileocecal valve; however, the section place in the jejunum was 5 cm from the duodenojejunal flexure to reach 50% of malabsorption. In the present research, the jejunojejunal anastomosis has been performed with the jejunum 10 cm from the duodenojejunal flexure and with the jejunum 60 cm from the ileocecal valve, to evaluate the histological alterations after the anastomosis, which is also used in the Roux-en-Y gastrojejunal derivation in human beings. The difference in the distance in the jejunal section place referred in studies is probably due to difference in animal weight; the rats used by Scudamore, C.H.e Freeman, H.J.^28^ weighed 140-160 g and in this study, they weighed on average 347 g. 

Xu et al.^36^ have described an experimental model of gastric derivation in rats. All the physiological principles that form the basis of the bariatric surgeries were used: restriction and malabsorption. It is the most faithful reproduction of the most frequent procedure used for treating obesity in humans; however, due to its complexity and the unavailability of mechanical stapling for anastomosis, it was not used in the present study.

Among the methods used to study the morphological alterations of the colonic mucosa, when highly exposed to bile salts, are: simple histological analysis^7^ ^,^ ^18^ ^,^ ^20^, counting of the tumor production in the small and large intestine subjected to induction with carcinogen^6,13,28^ and histology of the crypts^6^ ^,^ ^34^. It is important to highlight the evident relation between the metabolisms of the bile salts and the dynamic alteration of the colonic epithelium. Studies^2^ ^,^ ^6^ ^,^ ^16^ ^,^ ^22^ ^,^ ^28^ ^,^ ^33^ ^,^ ^35^ ^,^ ^37^ have reported that the malabsorptive surgeries have proliferative effects on the colonic mucosa. However, other studies^13^ ^,^ ^29^ ^,^ ^30^ disagree. In this study, it was not observed significant increase in the cell proliferation in the ascending colon in the experimental group after the jejunojejunal derivation. Sainsbury et al.^27^, however, have studied obese patients subjected to the Roux-en-Y gastric bypass and normal controls of BMI. Rectal biopsies were collected six months before and after the procedure. Prior to the procedure, all obese patients had a higher rectal epithelial cells mitosis counting (increased by 73%, p<0,01), bigger crypt area (increased by 36%, p<0,01) and the crypt branching was more than twice the normal in comparison with the control groups. However, unexpectedly, after the gastric bypass with significant weight loss there was a mitosis increase (75% higher than during the preoperative period, p=0,001) and apoptosis decrease (p=0,033). 

The importance of the relation between the cell proliferation and the carcinogenesis is emphasized^25^ ^,^ ^33^. It has been evaluated in patients with one or more colonic tumors in their previous medical history. Tumors from 106 sigmoid colons and 130 ascending colons from 246 patients were submitted to Ki-67 marker analysis to evaluate the proliferation. The authors have reported that patients with a higher Ki-67 marker index in the upper third in apparently normal sigmoid mucosa - but not in the ascending colon - presented more tumors with statistical significance in the control colonoscopy performed two years later. They concluded that the higher Ki-67 marker index in the middle third in the apparently normal mucosa of the sigmoid indicated high colorectal cancer risk. According to the model proposed by Fearon e Volgestein^9^, the colorectal carcinogenesis initiates from proliferative alterations and, subsequently, structural alterations on the mucosa arise. The role of the cell proliferation in the malignant transformation and the subsequent colorectal tumors growth is complex^11^ ^,^ ^23^. This study could not affirm indications of carcinogenesis because the p53 used as a tumor marker was negative in all cases and the cell proliferation in the experimental group´s ascending colon was lower than in the control group.

The evaluation of the cell proliferation is based on the production rate of the crypt cells using several methods^2^ ^,^ ^10^ ^,^ ^19^. In this study, the Ki-67 was used to evaluate this proliferation and no significant difference was noticed between the two groups regarding the expression of this marker in the jejunum and ileum. In the colon, there was a significant difference between the groups regarding the Ki-67 expression. This result suggests that the jejunojejunal anastomosis, which is performed in the Y-en-Roux gastrojejunal derivation keeping the common loop longer, suffers less with the aggression from the bile proliferative effects and reduced exposure to the bile salts than the jejunoileal shunts with shorter loops. 

Sylvan et al.^30^ have evaluated the colorectal cancer risk after the jejunoileal derivation in 30 women operated 11-17 years before. The biopsy material was taken from the cecum, ascending, transverse, descending colon and rectum. These samples were submitted to histological evaluation and to flow cytometric analysis for 49 DNA abnormalities. The results showed an increase in the proliferative activity of the colonic mucosa in the operated group. In the studies with animals, there was a wide variation in the period between the surgical procedure and the sacrifice: 32 weeks^13^, 30 weeks^6^ and only six weeks^28^. Despite the time variation, Kozoni et al.^16^ considered four weeks as the initial stages of the carcinogenesis. Unlike Teixeira´s study^31^, these authors evaluated the proliferation and apoptosis of the epithelial colonic cells after the rectal instillation of lithocholic acid; they reported increase in the crypt length compared with the one of the control group. In this study, the period of nine weeks between the surgical procedure and the animals’ death was chosen because it has been demonstrated by references to be enough to evaluate the presence of histological alterations related to the procedure. 

The right side of the colons (cecum and ascending colon) is the most susceptible to the bile proliferative effects for being more exposed to the bile salts^32^ ^,^ ^34^. Based on the cecum resistance to the cancer induction, compared with other colonic segments, the hypothesis of a cecum analogy to the human appendage has been raised. For these reasons, in another study^31^ the segment chosen for the analysis of the proliferative activity was the ascending colon, which also motivated its application in this research. 

Precise tissue markers allow a correlation between the neoplasia susceptibility and adjuvant therapies, fact not yet established. 

The limitation of this study was that, in fact, an ideal tumor marker - serum or tissue - is non-existent. The identification of efficient tumor markers will allow the establishment of an improved level of treatment for patients with malignant colorectal neoplasia. While more specific markers do not arise, and there is hope that they will in the near future, the understanding and rational use of the currently available markers can provide superior results in colon and rectum tumor therapy. 

## CONCLUSIONS

The jejunojejunal deviation performed in the Y-en-Roux gastrojejunal derivation predisposes proliferative effects causing an increase in the mucosa thickness, villi height and crypt depth of the jejunum, ileum and ascending colon.
